# 3-Benzyl-7-meth­oxy-9-phenyl-2-tosyl-2,3,3a,4,9,9a-hexa­hydro-1*H*-pyrrolo[3,4-*b*]quinoline

**DOI:** 10.1107/S1600536809044973

**Published:** 2009-10-31

**Authors:** K. Chinnakali, D. Sudha, M. Jayagobi, R. Raghunathan, Hoong-Kun Fun

**Affiliations:** a Department of Physics, Anna University Chennai, Chennai 600 025, India; b Department of Organic Chemistry, University of Madras, Guindy Campus, Chennai 600 025, India; c X-ray Crystallography Unit, School of Physics, Universiti Sains Malaysia, 11800 USM, Penang, Malaysia

## Abstract

In the title compound, C_32_H_32_N_2_O_3_S, the pyrrolidine ring adopts an envelope conformation with the methine C atom nearest to the phenyl ring as the flap atom. The tetra­hydro­pyridine ring has a half-chair conformation. The two rings are *trans*-fused. The phenyl ring bound to the tetra­hydro­pyridine is oriented almost perpendicular [dihedral angle = 86.35 (10)°] to the fused benzene ring. The dihedral angle between the benzyl­phenyl ring and the sulfonyl-bound phenyl ring is 69.43 (10)°. A very weak N—H⋯π inter­action is observed in the mol­ecular structure. In the crystal, mol­ecules translated one unit along the *b* axis are linked into *C*(10) chains by C—H⋯O hydrogen bonds; adjacent chains are linked *via* C—H⋯π inter­actions, forming a two-dimensional network parallel to the *bc* plane.

## Related literature

For biological activity of pyrroloquinoline derivatives, see: Ryu *et al.* (2009 ▶); Tsuji *et al.* (1995 ▶); Ferlin *et al.* (2001 ▶). For related structures, see: Sudha *et al.* (2007 ▶, 2008*a* ▶,*b* ▶). For ring puckering parameters, see: Cremer & Pople (1975 ▶). For asymmetry parameters, see: Duax *et al.* (1976 ▶).
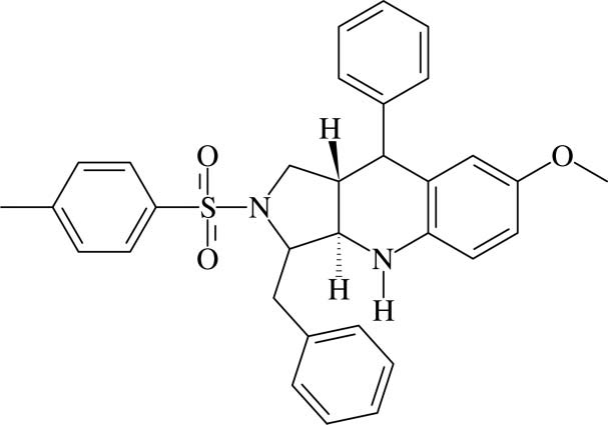


## Experimental

### 

#### Crystal data

C_32_H_32_N_2_O_3_S
*M* *_r_* = 524.66
Monoclinic, 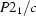

*a* = 21.5063 (9) Å
*b* = 11.6188 (5) Å
*c* = 10.7616 (4) Å
β = 98.219 (2)°
*V* = 2661.46 (19) Å^3^
*Z* = 4
Mo *K*α radiation
μ = 0.16 mm^−1^
*T* = 100 K
0.32 × 0.30 × 0.08 mm

#### Data collection

Bruker SMART APEXII CCD area-detector diffractometer
Absorption correction: multi-scan (*SADABS*; Bruker, 2005 ▶) *T*_min_ = 0.636, *T*_max_ = 0.987
28181 measured reflections
6088 independent reflections
4661 reflections with *I* > 2σ(*I*)
*R*_int_ = 0.057

#### Refinement

*R*[*F* ^2^ > 2σ(*F* ^2^)] = 0.054
*wR*(*F* ^2^) = 0.155
*S* = 1.02
6088 reflections
349 parameters
H atoms treated by a mixture of independent and constrained refinement
Δρ_max_ = 0.46 e Å^−3^
Δρ_min_ = −0.54 e Å^−3^

### 

Data collection: *APEX2* (Bruker, 2005 ▶); cell refinement: *SAINT* (Bruker, 2005 ▶); data reduction: *SAINT*; program(s) used to solve structure: *SHELXTL* (Sheldrick, 2008 ▶); program(s) used to refine structure: *SHELXTL*; molecular graphics: *SHELXTL*; software used to prepare material for publication: *SHELXTL* and *PLATON* (Spek, 2009 ▶).

## Supplementary Material

Crystal structure: contains datablocks global, I. DOI: 10.1107/S1600536809044973/lh2940sup1.cif

Structure factors: contains datablocks I. DOI: 10.1107/S1600536809044973/lh2940Isup2.hkl

Additional supplementary materials:  crystallographic information; 3D view; checkCIF report

## Figures and Tables

**Table 1:** Hydrogen-bond geometry (Å, °)

*D*—H⋯*A*	*D*—H	H⋯*A*	*D*⋯*A*	*D*—H⋯*A*
C30—H30⋯O3^i^	0.93	2.53	3.196 (3)	128
C24—H24⋯*Cg*1^ii^	0.93	2.56	3.476 (2)	169
N2—H1*N*2⋯*Cg*2	0.88 (2)	3.06	3.837 (2)	147

Symmetry codes: (i) 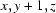; (ii) 
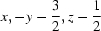
. *Cg*1 and *Cg*2 are the centroids of the C4–C9 and C26–C31 rings, respectively.

**Fig. 1.**
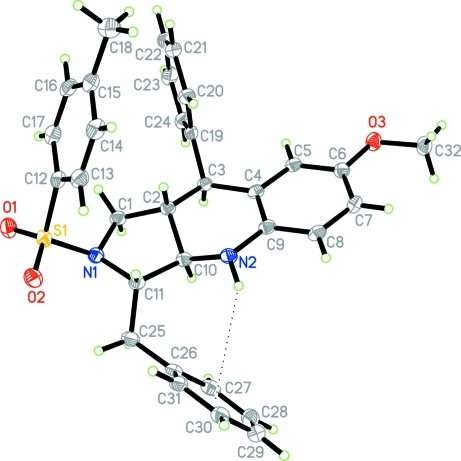
The molecular structure of the title compound. Displacement ellipsoids are drawn at the 50% probability level. The dotted line indicates an N—H···π interaction.

**Fig. 2.**
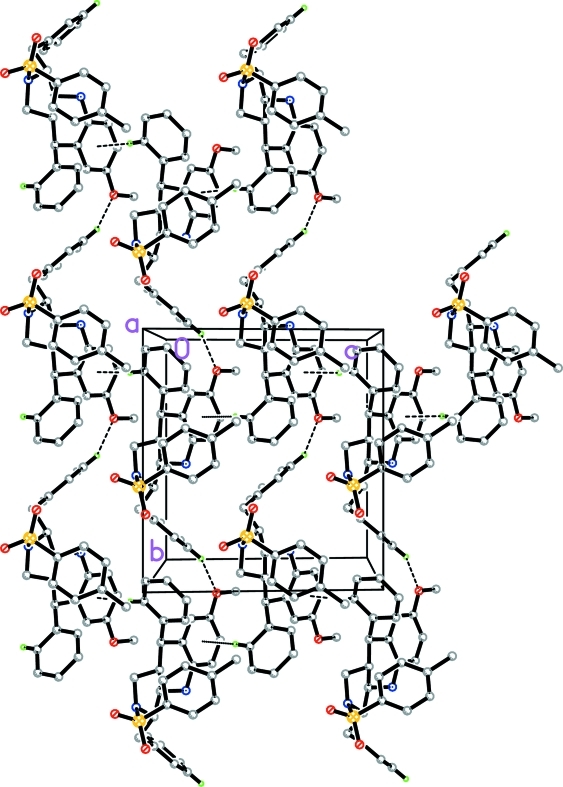
Crystal packing of the title compound. C—H···O hydrogen bonds and C—H···π interactions are shown as dashed lines. For the sake of clarity, H atoms not involved in the interactions have been omitted.

## supplementary crystallographic information

### Comment

Pyrroloquinoline compounds exhibit antifungal (Ryu *et al.*, 2009), antibacterial (Tsuji *et al.*, 1995) and antiproliferative (Ferlin *et al.*, 2001) activities. We report here the crystal structure of the title compound, a pyrrolo[3,4-*b*]quinoline derivative.

The pyrrolidine ring adopts an envelope conformation with C2 as the flap atom. Atom C2 deviates by 0.663 (3) Å from the plane passing through the other four atoms of the ring (r.m.s. deviation 0.020 Å). The asymmetry parameter (Duax *et al.*, 1976) ΔC_s_[C2] = 5.4 (2)° and the puckering parameters (Cremer & Pople, 1975) q_2_ = 0.439 (2) Å and φ = 78.2 (3)°. The tosyl group is attached to the pyrrolidine ring in a biaxial position. The tetrahydropyridine ring adopts a half-chair conformation with an asymmetry parameter ΔC_2_[C2—C10] of 7.6 (2)°. The phenyl group attached to the tetrahydropyridine ring is also in a biaxial position. The dihedral angle between the C4—C9 and C19—C24 rings is 86.35 (10)° and that between the C12—C17 and C26—C31 rings is 69.43 (10)°. A very weak N—H···π interaction (Table 1) is observed in the molecular structure. Bond lengths and angles are comparable with those observed in related structures (Sudha *et al.*, 2007,2008*a*,b).

In the crystal structure, molecules translated one unit along the *b* axis are linked into C(10) chains by C—H···O hydrogen bonds. Glide-related molecules in adjacent chains are linked *via* C—H···π interactions involving the C4—C9 ring, forming a two-dimensional network parallel to the *bc* plane (Fig. 2).

### Experimental

InCl_3_ (20 mol%) was added to a mixture of 2-(*N*-cinnamyl-*N*-tosylamino)-3-phenyl propanal (1 mmol) and *p*-methoxy aniline (1 mmol) in acetonitrile (20 ml). The reaction mixture was stirred at room temperature for 1 min. On completion of the reaction, as indicated by TLC, the mixture was quenched with water and extracted with ethyl acetate. The organic layer was washed with brine and dried over Na_2_SO_4_. The solvent was evaporated *in vacuo* and the crude product was chromatographed on silica gel using a hexane-ethyl acetate (8.5:1.5 *v*/*v*) mixture to obtain the title compound. The compound was recrystallized from ethyl acetate solution by slow evaporation.

### Refinement

The N-bound H atom was located in a difference map and refined freely [N—H = 0.88 (2) Å]. The remaining H atoms were positioned geometrically (C—H = 0.93–0.98 Å) and allowed to ride on their parent atoms, with *U*_iso_(H) = 1.2*U*_eq_(C) or 1.5*U*_eq_(C_methyl_). A rotating group model was used for methyl groups. Reflection 100 was partially obscured by the beam stop and was omitted.

### Crystal data

**Table:**

C_32_H_32_N_2_O_3_S	*F*(000) = 1112
*M**_r_* = 524.66	*D*_x_ = 1.309 Mg m^−^^3^
Monoclinic, *P*2_1_/*c*	Mo *K*α radiation, λ = 0.71073 Å
Hall symbol: -P 2ybc	Cell parameters from 6305 reflections
*a* = 21.5063 (9) Å	θ = 2.6–28.5°
*b* = 11.6188 (5) Å	µ = 0.16 mm^−^^1^
*c* = 10.7616 (4) Å	*T* = 100 K
β = 98.219 (2)°	Plate, colourless
*V* = 2661.46 (19) Å^3^	0.32 × 0.30 × 0.08 mm
*Z* = 4	

### Data collection

**Table:**

Bruker SMART APEXII CCD area-detector diffractometer	6088 independent reflections
Radiation source: fine-focus sealed tube	4661 reflections with *I* > 2σ(*I*)
graphite	*R*_int_ = 0.057
φ and ω scans	θ_max_ = 27.5°, θ_min_ = 2.0°
Absorption correction: multi-scan (*SADABS*; Bruker, 2005)	*h* = −23→27
*T*_min_ = 0.636, *T*_max_ = 0.987	*k* = −15→14
28181 measured reflections	*l* = −13→13

### Refinement

**Table:**

Refinement on *F*^2^	Primary atom site location: structure-invariant direct methods
Least-squares matrix: full	Secondary atom site location: difference Fourier map
*R*[*F*^2^ > 2σ(*F*^2^)] = 0.054	Hydrogen site location: inferred from neighbouring sites
*wR*(*F*^2^) = 0.155	H atoms treated by a mixture of independent and constrained refinement
*S* = 1.02	*w* = 1/[σ^2^(*F*_o_^2^) + (0.0891*P*)^2^ + 0.9992*P*] where *P* = (*F*_o_^2^ + 2*F*_c_^2^)/3
6088 reflections	(Δ/σ)_max_ = 0.001
349 parameters	Δρ_max_ = 0.46 e Å^−^^3^
0 restraints	Δρ_min_ = −0.54 e Å^−^^3^

### Special details

**Table:**

Geometry. All e.s.d.'s (except the e.s.d. in the dihedral angle between two l.s. planes) are estimated using the full covariance matrix. The cell e.s.d.'s are taken into account individually in the estimation of e.s.d.'s in distances, angles and torsion angles; correlations between e.s.d.'s in cell parameters are only used when they are defined by crystal symmetry. An approximate (isotropic) treatment of cell e.s.d.'s is used for estimating e.s.d.'s involving l.s. planes.
Refinement. Refinement of *F*^2^ against ALL reflections. The weighted *R*-factor *wR* and goodness of fit *S* are based on *F*^2^, conventional *R*-factors *R* are based on *F*, with *F* set to zero for negative *F*^2^. The threshold expression of *F*^2^ > σ(*F*^2^) is used only for calculating *R*-factors(gt) *etc*. and is not relevant to the choice of reflections for refinement. *R*-factors based on *F*^2^ are statistically about twice as large as those based on *F*, and *R*- factors based on ALL data will be even larger.

### Fractional atomic coordinates and isotropic or equivalent isotropic displacement parameters (Å^2^)

**Table:**

	*x*	*y*	*z*	*U*_iso_*/*U*_eq_	
S1	0.36811 (2)	0.64135 (4)	−0.09053 (5)	0.02080 (15)	
O1	0.39510 (7)	0.60291 (13)	−0.19725 (13)	0.0264 (3)	
O2	0.36734 (7)	0.76149 (12)	−0.06064 (14)	0.0266 (3)	
O3	0.07988 (7)	0.14332 (12)	0.25445 (14)	0.0235 (3)	
N1	0.29441 (8)	0.59952 (14)	−0.11262 (15)	0.0197 (4)	
N2	0.18416 (8)	0.55576 (14)	0.11176 (16)	0.0202 (4)	
H1N2	0.1626 (11)	0.619 (2)	0.122 (2)	0.024 (6)*	
C1	0.28306 (10)	0.47333 (16)	−0.12239 (18)	0.0195 (4)	
H1A	0.3222	0.4312	−0.1194	0.023*	
H1B	0.2557	0.4539	−0.1991	0.023*	
C2	0.25163 (9)	0.44865 (15)	−0.00752 (17)	0.0172 (4)	
H2	0.2834	0.4530	0.0673	0.021*	
C3	0.21612 (9)	0.33569 (15)	−0.00428 (17)	0.0169 (4)	
H3	0.1899	0.3268	−0.0860	0.020*	
C4	0.17183 (9)	0.34490 (16)	0.09480 (17)	0.0176 (4)	
C5	0.14457 (9)	0.24516 (16)	0.13452 (18)	0.0182 (4)	
H5	0.1546	0.1747	0.1016	0.022*	
C6	0.10267 (9)	0.24795 (16)	0.22227 (18)	0.0194 (4)	
C7	0.08772 (10)	0.35345 (17)	0.27171 (19)	0.0213 (4)	
H7	0.0602	0.3569	0.3308	0.026*	
C8	0.11424 (10)	0.45329 (17)	0.23202 (19)	0.0213 (4)	
H8	0.1037	0.5236	0.2648	0.026*	
C9	0.15627 (9)	0.45154 (16)	0.14423 (18)	0.0181 (4)	
C10	0.20811 (9)	0.55138 (16)	−0.00791 (18)	0.0179 (4)	
H10	0.1731	0.5435	−0.0764	0.022*	
C11	0.24967 (10)	0.65334 (16)	−0.03459 (18)	0.0192 (4)	
H11	0.2727	0.6828	0.0441	0.023*	
C12	0.40517 (9)	0.56541 (17)	0.04217 (18)	0.0206 (4)	
C13	0.40440 (10)	0.60984 (18)	0.16170 (19)	0.0231 (4)	
H13	0.3878	0.6825	0.1723	0.028*	
C14	0.42880 (10)	0.54439 (18)	0.2653 (2)	0.0249 (5)	
H14	0.4288	0.5744	0.3454	0.030*	
C15	0.45327 (10)	0.43488 (19)	0.2518 (2)	0.0251 (5)	
C16	0.45382 (10)	0.39268 (18)	0.1305 (2)	0.0238 (4)	
H16	0.4702	0.3199	0.1197	0.029*	
C17	0.43044 (10)	0.45702 (17)	0.0261 (2)	0.0233 (4)	
H17	0.4316	0.4281	−0.0541	0.028*	
C18	0.47876 (12)	0.3636 (2)	0.3642 (2)	0.0334 (5)	
H18A	0.4656	0.3963	0.4382	0.050*	
H18B	0.5238	0.3627	0.3732	0.050*	
H18C	0.4631	0.2864	0.3531	0.050*	
C19	0.26098 (9)	0.23397 (16)	0.01226 (18)	0.0177 (4)	
C20	0.30745 (10)	0.22449 (17)	0.11657 (19)	0.0220 (4)	
H20	0.3105	0.2803	0.1791	0.026*	
C21	0.34910 (10)	0.13259 (18)	0.1278 (2)	0.0254 (5)	
H21	0.3800	0.1277	0.1974	0.030*	
C22	0.34495 (11)	0.04784 (17)	0.0358 (2)	0.0268 (5)	
H22	0.3725	−0.0143	0.0441	0.032*	
C23	0.29953 (11)	0.05698 (17)	−0.0680 (2)	0.0269 (5)	
H23	0.2968	0.0013	−0.1306	0.032*	
C24	0.25761 (10)	0.14916 (17)	−0.0797 (2)	0.0223 (4)	
H24	0.2270	0.1540	−0.1499	0.027*	
C25	0.21481 (10)	0.75164 (17)	−0.10939 (19)	0.0227 (4)	
H25A	0.2447	0.8112	−0.1230	0.027*	
H25B	0.1962	0.7229	−0.1909	0.027*	
C26	0.16400 (10)	0.80340 (16)	−0.04408 (18)	0.0202 (4)	
C27	0.10099 (10)	0.77711 (17)	−0.0814 (2)	0.0249 (5)	
H27	0.0900	0.7246	−0.1461	0.030*	
C28	0.05413 (11)	0.82820 (19)	−0.0235 (2)	0.0276 (5)	
H28	0.0122	0.8108	−0.0503	0.033*	
C29	0.07005 (11)	0.90497 (18)	0.0741 (2)	0.0275 (5)	
H29	0.0388	0.9400	0.1124	0.033*	
C30	0.13279 (10)	0.92955 (17)	0.1147 (2)	0.0237 (4)	
H30	0.1437	0.9797	0.1817	0.028*	
C31	0.17939 (10)	0.87961 (16)	0.05583 (19)	0.0213 (4)	
H31	0.2213	0.8971	0.0832	0.026*	
C32	0.03712 (10)	0.14275 (18)	0.3447 (2)	0.0255 (5)	
H32A	0.0236	0.0653	0.3568	0.038*	
H32B	0.0014	0.1897	0.3150	0.038*	
H32C	0.0577	0.1728	0.4230	0.038*	

### Atomic displacement parameters (Å^2^)

**Table:**

	*U*^11^	*U*^22^	*U*^33^	*U*^12^	*U*^13^	*U*^23^
S1	0.0206 (3)	0.0180 (3)	0.0242 (3)	−0.00236 (19)	0.0046 (2)	0.00132 (18)
O1	0.0265 (9)	0.0282 (8)	0.0259 (8)	−0.0026 (6)	0.0091 (6)	0.0015 (6)
O2	0.0252 (9)	0.0192 (7)	0.0353 (8)	−0.0044 (6)	0.0042 (7)	0.0022 (6)
O3	0.0211 (8)	0.0197 (7)	0.0313 (8)	−0.0037 (6)	0.0089 (6)	−0.0016 (6)
N1	0.0190 (9)	0.0161 (8)	0.0245 (9)	0.0001 (7)	0.0044 (7)	0.0006 (6)
N2	0.0250 (10)	0.0124 (8)	0.0243 (9)	0.0029 (7)	0.0075 (7)	−0.0008 (6)
C1	0.0199 (11)	0.0162 (9)	0.0222 (10)	0.0009 (7)	0.0029 (8)	−0.0006 (7)
C2	0.0164 (10)	0.0145 (9)	0.0204 (9)	0.0006 (7)	0.0014 (8)	−0.0007 (7)
C3	0.0156 (10)	0.0159 (9)	0.0189 (9)	0.0004 (7)	0.0015 (7)	−0.0002 (7)
C4	0.0150 (10)	0.0182 (9)	0.0188 (9)	0.0020 (7)	−0.0007 (7)	−0.0015 (7)
C5	0.0161 (10)	0.0155 (9)	0.0223 (10)	0.0012 (7)	0.0008 (8)	−0.0015 (7)
C6	0.0154 (10)	0.0179 (9)	0.0242 (10)	−0.0018 (7)	0.0007 (8)	0.0005 (7)
C7	0.0158 (10)	0.0246 (10)	0.0245 (10)	0.0012 (8)	0.0056 (8)	−0.0012 (8)
C8	0.0189 (11)	0.0180 (10)	0.0270 (10)	0.0039 (8)	0.0030 (8)	−0.0035 (8)
C9	0.0144 (10)	0.0169 (9)	0.0222 (10)	0.0002 (7)	−0.0009 (8)	−0.0010 (7)
C10	0.0182 (10)	0.0163 (9)	0.0190 (9)	0.0006 (7)	0.0017 (8)	−0.0003 (7)
C11	0.0196 (11)	0.0159 (9)	0.0224 (10)	0.0010 (7)	0.0038 (8)	−0.0009 (7)
C12	0.0152 (10)	0.0221 (10)	0.0248 (10)	−0.0020 (8)	0.0038 (8)	0.0008 (8)
C13	0.0208 (11)	0.0205 (10)	0.0282 (11)	−0.0011 (8)	0.0045 (8)	−0.0022 (8)
C14	0.0212 (11)	0.0296 (11)	0.0239 (10)	−0.0007 (9)	0.0035 (8)	−0.0036 (8)
C15	0.0151 (11)	0.0296 (11)	0.0301 (11)	−0.0011 (8)	0.0022 (8)	0.0031 (9)
C16	0.0151 (10)	0.0226 (10)	0.0336 (11)	0.0003 (8)	0.0035 (8)	−0.0014 (8)
C17	0.0205 (11)	0.0236 (10)	0.0266 (11)	−0.0016 (8)	0.0058 (8)	−0.0043 (8)
C18	0.0320 (14)	0.0355 (13)	0.0325 (12)	0.0057 (10)	0.0036 (10)	0.0063 (10)
C19	0.0160 (10)	0.0147 (9)	0.0235 (10)	−0.0002 (7)	0.0068 (8)	0.0028 (7)
C20	0.0225 (11)	0.0212 (10)	0.0228 (10)	0.0027 (8)	0.0046 (8)	0.0006 (8)
C21	0.0212 (11)	0.0280 (11)	0.0277 (11)	0.0056 (9)	0.0058 (9)	0.0086 (8)
C22	0.0267 (12)	0.0169 (10)	0.0399 (12)	0.0061 (8)	0.0152 (10)	0.0061 (8)
C23	0.0274 (12)	0.0169 (10)	0.0386 (12)	−0.0010 (8)	0.0127 (10)	−0.0058 (9)
C24	0.0199 (11)	0.0197 (10)	0.0277 (11)	−0.0021 (8)	0.0047 (8)	−0.0026 (8)
C25	0.0269 (12)	0.0178 (10)	0.0237 (10)	0.0034 (8)	0.0044 (9)	0.0038 (8)
C26	0.0241 (11)	0.0126 (9)	0.0238 (10)	0.0023 (7)	0.0032 (8)	0.0059 (7)
C27	0.0287 (12)	0.0176 (9)	0.0271 (11)	−0.0024 (8)	−0.0006 (9)	0.0027 (8)
C28	0.0189 (11)	0.0301 (11)	0.0331 (12)	−0.0022 (9)	0.0011 (9)	0.0079 (9)
C29	0.0248 (12)	0.0253 (11)	0.0342 (12)	0.0049 (9)	0.0099 (9)	0.0075 (9)
C30	0.0280 (12)	0.0173 (9)	0.0264 (10)	0.0005 (8)	0.0057 (9)	0.0021 (8)
C31	0.0193 (11)	0.0158 (9)	0.0282 (11)	0.0005 (8)	0.0015 (8)	0.0038 (8)
C32	0.0210 (11)	0.0261 (11)	0.0307 (11)	−0.0041 (8)	0.0080 (9)	0.0006 (9)

### Geometric parameters (Å, °)

**Table:**

S1—O1	1.4303 (15)	C14—H14	0.93
S1—O2	1.4332 (15)	C15—C16	1.396 (3)
S1—N1	1.6424 (18)	C15—C18	1.504 (3)
S1—C12	1.768 (2)	C16—C17	1.383 (3)
O3—C6	1.374 (2)	C16—H16	0.93
O3—C32	1.430 (2)	C17—H17	0.93
N1—C1	1.488 (2)	C18—H18A	0.96
N1—C11	1.501 (2)	C18—H18B	0.96
N2—C9	1.417 (2)	C18—H18C	0.96
N2—C10	1.455 (2)	C19—C24	1.391 (3)
N2—H1N2	0.88 (2)	C19—C20	1.396 (3)
C1—C2	1.519 (3)	C20—C21	1.388 (3)
C1—H1A	0.97	C20—H20	0.93
C1—H1B	0.97	C21—C22	1.390 (3)
C2—C10	1.516 (3)	C21—H21	0.93
C2—C3	1.521 (3)	C22—C23	1.379 (3)
C2—H2	0.98	C22—H22	0.93
C3—C19	1.520 (3)	C23—C24	1.394 (3)
C3—C4	1.532 (3)	C23—H23	0.93
C3—H3	0.98	C24—H24	0.93
C4—C5	1.393 (3)	C25—C26	1.506 (3)
C4—C9	1.407 (3)	C25—H25A	0.97
C5—C6	1.396 (3)	C25—H25B	0.97
C5—H5	0.93	C26—C27	1.391 (3)
C6—C7	1.392 (3)	C26—C31	1.396 (3)
C7—C8	1.387 (3)	C27—C28	1.392 (3)
C7—H7	0.93	C27—H27	0.93
C8—C9	1.398 (3)	C28—C29	1.383 (3)
C8—H8	0.93	C28—H28	0.93
C10—C11	1.536 (3)	C29—C30	1.387 (3)
C10—H10	0.98	C29—H29	0.93
C11—C25	1.530 (3)	C30—C31	1.387 (3)
C11—H11	0.98	C30—H30	0.93
C12—C13	1.388 (3)	C31—H31	0.93
C12—C17	1.392 (3)	C32—H32A	0.96
C13—C14	1.389 (3)	C32—H32B	0.96
C13—H13	0.93	C32—H32C	0.96
C14—C15	1.392 (3)		
			
O1—S1—O2	120.22 (9)	C13—C14—C15	121.46 (19)
O1—S1—N1	106.58 (9)	C13—C14—H14	119.3
O2—S1—N1	106.18 (9)	C15—C14—H14	119.3
O1—S1—C12	107.76 (9)	C14—C15—C16	118.19 (19)
O2—S1—C12	108.85 (9)	C14—C15—C18	121.25 (19)
N1—S1—C12	106.47 (9)	C16—C15—C18	120.6 (2)
C6—O3—C32	117.56 (15)	C17—C16—C15	121.27 (19)
C1—N1—C11	109.76 (15)	C17—C16—H16	119.4
C1—N1—S1	116.56 (13)	C15—C16—H16	119.4
C11—N1—S1	118.58 (13)	C16—C17—C12	119.42 (19)
C9—N2—C10	113.85 (15)	C16—C17—H17	120.3
C9—N2—H1N2	115.6 (15)	C12—C17—H17	120.3
C10—N2—H1N2	113.5 (15)	C15—C18—H18A	109.5
N1—C1—C2	102.50 (15)	C15—C18—H18B	109.5
N1—C1—H1A	111.3	H18A—C18—H18B	109.5
C2—C1—H1A	111.3	C15—C18—H18C	109.5
N1—C1—H1B	111.3	H18A—C18—H18C	109.5
C2—C1—H1B	111.3	H18B—C18—H18C	109.5
H1A—C1—H1B	109.2	C24—C19—C20	118.32 (18)
C10—C2—C1	101.27 (15)	C24—C19—C3	120.00 (18)
C10—C2—C3	111.56 (16)	C20—C19—C3	121.65 (17)
C1—C2—C3	117.70 (15)	C21—C20—C19	120.63 (19)
C10—C2—H2	108.6	C21—C20—H20	119.7
C1—C2—H2	108.6	C19—C20—H20	119.7
C3—C2—H2	108.6	C20—C21—C22	120.5 (2)
C19—C3—C2	111.18 (16)	C20—C21—H21	119.7
C19—C3—C4	114.85 (15)	C22—C21—H21	119.7
C2—C3—C4	108.65 (15)	C23—C22—C21	119.24 (19)
C19—C3—H3	107.3	C23—C22—H22	120.4
C2—C3—H3	107.3	C21—C22—H22	120.4
C4—C3—H3	107.3	C22—C23—C24	120.41 (19)
C5—C4—C9	118.88 (18)	C22—C23—H23	119.8
C5—C4—C3	119.10 (16)	C24—C23—H23	119.8
C9—C4—C3	121.99 (17)	C19—C24—C23	120.9 (2)
C4—C5—C6	121.94 (17)	C19—C24—H24	119.6
C4—C5—H5	119.0	C23—C24—H24	119.6
C6—C5—H5	119.0	C26—C25—C11	112.71 (16)
O3—C6—C7	124.94 (18)	C26—C25—H25A	109.1
O3—C6—C5	115.99 (17)	C11—C25—H25A	109.1
C7—C6—C5	119.07 (18)	C26—C25—H25B	109.1
C8—C7—C6	119.42 (18)	C11—C25—H25B	109.1
C8—C7—H7	120.3	H25A—C25—H25B	107.8
C6—C7—H7	120.3	C27—C26—C31	118.40 (19)
C7—C8—C9	122.02 (18)	C27—C26—C25	121.28 (19)
C7—C8—H8	119.0	C31—C26—C25	120.32 (19)
C9—C8—H8	119.0	C26—C27—C28	121.0 (2)
C8—C9—C4	118.68 (17)	C26—C27—H27	119.5
C8—C9—N2	119.44 (17)	C28—C27—H27	119.5
C4—C9—N2	121.81 (17)	C29—C28—C27	119.9 (2)
N2—C10—C2	108.76 (15)	C29—C28—H28	120.0
N2—C10—C11	115.32 (16)	C27—C28—H28	120.0
C2—C10—C11	103.43 (16)	C28—C29—C30	119.7 (2)
N2—C10—H10	109.7	C28—C29—H29	120.1
C2—C10—H10	109.7	C30—C29—H29	120.1
C11—C10—H10	109.7	C31—C30—C29	120.2 (2)
N1—C11—C25	108.76 (15)	C31—C30—H30	119.9
N1—C11—C10	102.71 (14)	C29—C30—H30	119.9
C25—C11—C10	114.82 (17)	C30—C31—C26	120.7 (2)
N1—C11—H11	110.1	C30—C31—H31	119.7
C25—C11—H11	110.1	C26—C31—H31	119.7
C10—C11—H11	110.1	O3—C32—H32A	109.5
C13—C12—C17	120.53 (19)	O3—C32—H32B	109.5
C13—C12—S1	119.93 (16)	H32A—C32—H32B	109.5
C17—C12—S1	119.33 (15)	O3—C32—H32C	109.5
C12—C13—C14	119.12 (19)	H32A—C32—H32C	109.5
C12—C13—H13	120.4	H32B—C32—H32C	109.5
C14—C13—H13	120.4		
			
O1—S1—N1—C1	61.38 (15)	N2—C10—C11—N1	148.55 (16)
O2—S1—N1—C1	−169.33 (13)	C2—C10—C11—N1	29.94 (19)
C12—S1—N1—C1	−53.44 (16)	N2—C10—C11—C25	−93.6 (2)
O1—S1—N1—C11	−163.96 (13)	C2—C10—C11—C25	147.81 (16)
O2—S1—N1—C11	−34.67 (16)	O1—S1—C12—C13	158.49 (16)
C12—S1—N1—C11	81.22 (15)	O2—S1—C12—C13	26.59 (19)
C11—N1—C1—C2	−23.1 (2)	N1—S1—C12—C13	−87.49 (18)
S1—N1—C1—C2	115.35 (15)	O1—S1—C12—C17	−26.68 (19)
N1—C1—C2—C10	40.98 (18)	O2—S1—C12—C17	−158.58 (16)
N1—C1—C2—C3	162.85 (16)	N1—S1—C12—C17	87.34 (18)
C10—C2—C3—C19	−173.02 (15)	C17—C12—C13—C14	−0.4 (3)
C1—C2—C3—C19	70.6 (2)	S1—C12—C13—C14	174.34 (16)
C10—C2—C3—C4	−45.7 (2)	C12—C13—C14—C15	−0.8 (3)
C1—C2—C3—C4	−162.10 (16)	C13—C14—C15—C16	1.2 (3)
C19—C3—C4—C5	−40.9 (2)	C13—C14—C15—C18	−179.1 (2)
C2—C3—C4—C5	−166.12 (17)	C14—C15—C16—C17	−0.4 (3)
C19—C3—C4—C9	141.09 (18)	C18—C15—C16—C17	179.9 (2)
C2—C3—C4—C9	15.9 (2)	C15—C16—C17—C12	−0.8 (3)
C9—C4—C5—C6	−0.4 (3)	C13—C12—C17—C16	1.2 (3)
C3—C4—C5—C6	−178.46 (18)	S1—C12—C17—C16	−173.61 (16)
C32—O3—C6—C7	0.6 (3)	C2—C3—C19—C24	−118.83 (19)
C32—O3—C6—C5	179.61 (18)	C4—C3—C19—C24	117.29 (19)
C4—C5—C6—O3	−179.17 (17)	C2—C3—C19—C20	59.2 (2)
C4—C5—C6—C7	−0.1 (3)	C4—C3—C19—C20	−64.7 (2)
O3—C6—C7—C8	179.58 (19)	C24—C19—C20—C21	0.0 (3)
C5—C6—C7—C8	0.6 (3)	C3—C19—C20—C21	−178.04 (18)
C6—C7—C8—C9	−0.6 (3)	C19—C20—C21—C22	−0.5 (3)
C7—C8—C9—C4	0.1 (3)	C20—C21—C22—C23	1.0 (3)
C7—C8—C9—N2	−177.01 (19)	C21—C22—C23—C24	−1.0 (3)
C5—C4—C9—C8	0.4 (3)	C20—C19—C24—C23	0.0 (3)
C3—C4—C9—C8	178.37 (17)	C3—C19—C24—C23	178.09 (18)
C5—C4—C9—N2	177.43 (18)	C22—C23—C24—C19	0.5 (3)
C3—C4—C9—N2	−4.6 (3)	N1—C11—C25—C26	174.16 (16)
C10—N2—C9—C8	−159.30 (18)	C10—C11—C25—C26	59.8 (2)
C10—N2—C9—C4	23.6 (3)	C11—C25—C26—C27	−102.6 (2)
C9—N2—C10—C2	−53.1 (2)	C11—C25—C26—C31	77.6 (2)
C9—N2—C10—C11	−168.66 (16)	C31—C26—C27—C28	2.0 (3)
C1—C2—C10—N2	−167.42 (15)	C25—C26—C27—C28	−177.81 (18)
C3—C2—C10—N2	66.5 (2)	C26—C27—C28—C29	−1.1 (3)
C1—C2—C10—C11	−44.36 (18)	C27—C28—C29—C30	−0.8 (3)
C3—C2—C10—C11	−170.41 (15)	C28—C29—C30—C31	1.6 (3)
C1—N1—C11—C25	−126.22 (17)	C29—C30—C31—C26	−0.5 (3)
S1—N1—C11—C25	96.32 (18)	C27—C26—C31—C30	−1.2 (3)
C1—N1—C11—C10	−4.1 (2)	C25—C26—C31—C30	178.62 (18)
S1—N1—C11—C10	−141.61 (14)		

### Hydrogen-bond geometry (Å, °)

**Table:**

*D*—H···*A*	*D*—H	H···*A*	*D*···*A*	*D*—H···*A*
C30—H30···O3^i^	0.93	2.53	3.196 (3)	128
C24—H24···Cg1^ii^	0.93	2.56	3.476 (2)	169
N2—H1N2···Cg2	0.88 (2)	3.06	3.837 (2)	147

Symmetry codes: (i) *x*, *y*+1, *z*; (ii) *x*, −*y*−3/2, *z*−1/2.
